# Feasibility of Implementing an Electronic Patient-Reported Outcome-Based Digital Health Platform in Pediatric Oncology and Palliative Care: Insights From the MyPal4Kids Multicenter Observational Study

**DOI:** 10.2196/80718

**Published:** 2026-08-11

**Authors:** Marcel Meyerheim, Lenka Radová, Anna Burns-Gebhart, Marvin Mergen, Pantelis Natsiavas, Panos Bonotis, Christina Karamanidou, Sheila Payne, Julia Downing, Julie Ling, Annette Sander, Kasra Mirzaie, Susan Dokutur, Jana Vaculíková, Petr Lokaj, Ondřej Rohleder, Norbert Graf

**Affiliations:** 1 Department of Pediatric Oncology and Hematology, Faculty of Medicine, Saarland University, Building 9, Homburg, 66421, Germany, 49 68411628045; 2 Center of Molecular Medicine, Central European Institute of Technology, Masaryk University, Brno, Czech Republic; 3 Department for Pediatric Oncology and Hematology, Hannover Medical School, Hannover, Germany; 4 Institute of Applied Biosciences, Centre for Research and Technology Hellas, Thessaloniki, Greece; 5 International Observatory on End of Life Care, Division of Health Research, Lancaster University, Lancaster, United Kingdom; 6 International Children's Palliative Care Network, Bristol, United Kingdom; 7 European Association for Palliative Care, Vilvoorde, Belgium; 8 Department of Pediatric Oncology, University Hospital Brno, Brno, Czech Republic

**Keywords:** electronic patient-reported outcomes, digital health, serious game, gamification, pediatric oncology, palliative care, patient engagement, mHealth, mobile health

## Abstract

**Background:**

Digital health solutions that incorporate electronic patient-reported outcomes (ePROs) hold promise for enhancing communication and patient engagement in pediatric oncology and palliative care. While ePROs are increasingly used in adult populations, their use in children, especially when combined with gamification, remains underexplored.

**Objective:**

This MyPal4Kids study aimed to assess the feasibility, acceptability, and user engagement of a digital health platform that integrates ePROs and a serious game, designed for pediatric oncology patients aged 6‐17 years and their caregivers.

**Methods:**

A multicenter observational feasibility study was conducted between December 2020 and September 2022 at 3 clinical sites in Germany and the Czech Republic. A total of 83 children and adolescents with cancer, their parent or legal guardian, as well as 10 health care professionals (HCPs) participated. Primary outcomes included platform acceptability and engagement, assessed through recruitment, participation, and attrition rates, adherence, and usability ratings. Secondary outcomes focused on the feasibility of ePRO-based data capture and the perceived impact on HCP workflows and communication. A mixed methods design was used, combining in-app data and surveys with qualitative insights from focus groups.

**Results:**

The recruitment rate was 55%, with an attrition rate of 18%. Usability was rated positively, with most users finding the platform intuitive. Sustained engagement declined over time, particularly among older children who found the serious game insufficiently engaging. Adherence varied by age group and HCP involvement. Participants reported increased motivation when symptom reports were acknowledged by HCPs, although the impact on communication was perceived inconsistently.

**Conclusions:**

The developed platform shows potential for use in clinical care and research. However, maintaining long-term engagement remains a challenge. Tailoring content to different age groups and strengthening feedback mechanisms from HCPs are critical for improving the user experience. Successful implementation in routine clinical practice will require integration into existing workflows and digital infrastructure, along with ongoing user-driven development.

## Introduction

The illness trajectory of children and adolescents with cancer presents a heavy burden for patients and their families, marked by diagnosis, the difficult treatment process, and fear of relapse or death. Physical and psychological challenges affect patients, relatives, and peers [1-3]. This underscores the need for comprehensive support, including palliative care, which should begin at diagnosis, not just at the end of life [4].

Recognizing this, pediatric oncology has shifted toward investigating patient-reported outcomes, typically collected via standardized questionnaires. Electronic patient-reported outcomes (ePROs) are digital versions, often available through mobile apps [5-7]. This patient-centered approach allows young patients with cancer to self-report experiences, preferences, concerns, and symptoms in real time [8], without relying on health care professionals (HCPs) [9]. Such patient-centered approaches aim to improve self-efficacy and health-related quality of life (QoL) for young patients with cancer and their relatives [9,10]. They also aim to improve communication among all stakeholders [11], addressing time constraints during appointments and providing continuous, nuanced information on patients’ well-being [12].

While ePROs have demonstrated utility as outcome measures in clinical trials involving childhood cancer and palliative care [6,13-17], their implementation in daily clinical practice remains limited and underexplored [13,18-22]. Expert guidance also highlights the need for carefully designed development and implementation processes for pediatric ePRO systems to ensure usability, clinical relevance, and successful integration into routine care [23-25].

Approaches such as serious games [26] and gamification [27-29] have gained increasing attention as strategies to enhance engagement with digital health interventions, particularly in younger populations [30-33]. Gamification elements (eg, rewards, narratives, or interactive tasks) may foster motivation, adherence, and sustained use of health apps, thereby potentially improving data completeness and the overall effectiveness of ePRO systems in supporting coping, communication, and self-expression. However, the use of gamification to engage young patients is still poorly understood [18,34-40].

At the same time, the application of digital health tools in pediatric oncology requires careful consideration of the heterogeneity of the target population. Children and adolescents differ widely in terms of cognitive development, emotional needs, and digital literacy, and their experiences of cancer vary depending on diagnosis, treatment intensity, and prognosis [1-3]. Moreover, the involvement of parents or caregivers plays a central role in treatment-related decision-making, distinguishing pediatric settings from adult care, while also being associated with increased levels of distress among caregivers [41,42]. These factors highlight the importance of designing flexible, developmentally appropriate platforms that can accommodate both self-reporting by patients and proxy reporting by caregivers, while remaining sensitive to the diverse clinical and psychosocial contexts within pediatric oncology.

The European Union-funded MyPal project (grant 825872 [43]) addressed these challenges by developing and evaluating a novel digital health platform under real-world conditions. The MyPal digital health platform and its components were developed by the MyPal project partners Foundation for Research and Technology-Hellas and Promotion Software GmbH. It encompassed two clinical studies: MyPal Adults (adult patients with cancer) [44] and MyPal4Kids (children and adolescents with cancer) [45]. MyPal4Kids aimed to evaluate the acceptability, appropriateness, and impact of an ePRO-based platform for pediatric oncology and palliative care, as perceived by children and adolescents with cancer, parents, and HCPs. The study also sought to identify barriers and facilitators relevant to the platform’s integration into routine care. An interdisciplinary team, including experts from pediatric oncology, palliative care, psychology, IT, and software development, oversaw all stages from design to implementation and evaluation.

Previous publications from the MyPal consortium have addressed key feasibility aspects [46], including a systematic review on ePRO interventions in palliative care [47], ethical and legal considerations [48], implementation frameworks [49], technical development [50], psycho-oncological aspects [51], and policy implications [52]. This paper presents the core findings of the MyPal4Kids clinical study, combining quantitative and qualitative data to evaluate whether integrating ePROs via a digital platform can support more personalized and sustainable care in pediatric oncology and palliative care.

## Methods

### Overview

The multicenter observational feasibility study, MyPal4Kids, was conducted from December 2020 to September 2022 at Saarland University (USAAR), Hannover Medical School (MHH; Germany), and University Hospital Brno (FNBRNO; Czech Republic). Recruitment closed on March 31, 2022 (last patient, first visit).

### Study Design

An observational study design was chosen to evaluate the feasibility of the platform in a real-world setting without randomization, enabling a naturalistic assessment of acceptability and engagement.

### Setting and Participants

The study took place in pediatric oncology units at all 3 participating hospitals, which provide inpatient and outpatient care to about 290 new pediatric oncology patients annually, supported by multidisciplinary teams. Eligible participants were children and adolescents with cancer aged 6-17 years diagnosed with solid tumors or leukemia and treated at one of the sites within the previous 12 months.

### Recruitment

Trained clinical staff recruited participants. Eligible patients and at least 1 parent or legal guardian were invited to participate in the 6-month study during an informational session. Written informed consent was obtained from patients and their parents. Study staff created user accounts, provided leaflets with QR codes for app installation and baseline questionnaires, and offered support as needed. Details of recruitment and eligibility are available in the protocol [45].

### Intervention: The MyPal Digital Health Platform

In addition to standard care, participants used the ePRO-based platform during their 6-month study participation. The platform had 3 components (Table 1) [50], which were available in German and Czech with a “read-aloud” functionality for younger users. A video demonstrating the MyPal platform and its apps is available online [53]. The platform was implemented through these 3 components and was offered for use within clinical workflows across user-selected devices. Participation was voluntary, and no mandatory actions or response pathways were established for HCPs. Data were securely transmitted and made available to HCPs to support clinical decision-making.

**Table 1.** A description of the 3 components of the MyPal digital health platform evaluated in MyPal4Kids.

Component	Description
MyPal-Child App	A mobile video game designed for young patients with cancer that integrates ePRO^a^-based symptom self-reporting and diary entries into the serious game “AquaScouts.” [50] The game immerses players in an underwater adventure in which infected areas are progressively restored through the gameplay. Players collect tokens while responding to prioritized symptom-related questions presented both inside and outside the game environment. Diary entries could be optionally shared with treating HCPs^b^ through the App. Gamification elements included obstacle avoidance mechanics associated with point deductions, selectable movement speeds, token-based rewards (eg, artifacts), daily high-score lists among study participants, and avatar customization intended to support motivation and engagement.
MyPal-Carer App	Enables parents to proxy-report their child’s symptoms and access relevant web links. Both this and the MyPal-Child App could be installed on mobile devices and were also used to complete study questionnaires.
Browser-based HCP interface	A web-accessible tool (via smartphone, tablet, or desktop) that allows real-time review of reported symptoms and diary entries through interactive visualizations. HCPs could add treatment protocols, symptoms, and reactions based on observed data. The study protocol did not prescribe specific HCP responses. The interface was used on a discretionary basis.

a ePRO: electronic patient-reported outcome.

b HCP: health care professional.

### Participatory Design

A participatory design approach guided the development of the platform with the aim to enhance its relevance, utility, and usability for the target population. This process included a literature review, prestudy focus groups [54], and structured discussions with patients, parents, and HCPs to identify needs, preferences, potential barriers, and desired functionalities. These activities served as a prestudy assessment to inform key design decisions and ensure that the platform was aligned with user requirements prior to implementation. Development followed an iterative feedback loop and included a 2-week pilot phase with children prior to study initiation to further refine the platform, identify technical issues, evaluate the local technical infrastructure, and obtain initial insights before the feasibility study commenced [50].

### Outcomes Measures

Participants were invited to use the platform and its apps for 6 months. Table 2 summarizes the study end points, outcome measures, and time points adapted from the published study protocol [45]. In addition to optional symptom reporting, patients and parents completed ePRO-based secondary end point measures via the apps. The questionnaires were selected based on validity, dissemination, digital availability, and author agreement (refer to Table 2 for end points and the assessment schedule and S5 in Multimedia Appendix 1 for questionnaire dimensions, rating scales, and scoring information).

**Table 2.** The study end points and outcome measures in MyPal4Kids.

Outcome measures and ePROs^a^	Time point of measure
Primary end points	
Acceptability of and engagement with the MyPal Platform	
Recruitment rate, participation rate, attrition rate, and adherence to the different components of the MyPal platform	M1-M6^b^ continuously
System Usability (validated System Usability Scale [SUS] [55]) and a self-adapted version for children (S1 in Multimedia Appendix 1, child-adapted SUS version)	Once after M6
Focus groups with children and their parents	Once after M6
Secondary end points	
Demonstrating the feasibility of measuring	
Children’s symptom burden (digital adaptation of the validated Mini-SSPedi^c^ [56] and SSPedi [57]): higher ratings indicate higher burden	M1-M6 optional anytime
Children’s QoL^d^ (validated PedsQL^e^ 3.0 Cancer Module [58,59]): higher scores indicate fewer problems and better QoL	Monthly (Baseline, M1-M6)
Parents’ satisfaction with cancer care (validated EORTC PATSAT-C33^f^ [60,61], adapted to assess parent’s satisfaction with children’s cancer care): higher scores indicate greater satisfaction with care	Monthly (Baseline, M1-M6)
Impact of pediatric illness on the family (validated IOFS^g^ [62,63]): higher scores indicate greater negative impact on family	Monthly (Baseline, M1-M6)
Parents’ QoL (EuroQol EQ-5D-3L [64]): higher visual analog scale scores indicate better perceived health	Monthly (Baseline, M1-M6)
Focus groups with children and their parents	Once after M6
Impact on HCPs^h^ due to the integration of ePROs in care	
Web-based follow-up questionnaire (S2 in Multimedia Appendix 1, questionnaire for HCPs)	Once after M6
Focus groups with project-internal and external HCPs involved in the study	Once after M6

a ePRO: electronic patient-reported outcome.

b M[X]: Month X of study participation.

c SSPedi: Symptom Screening in Pediatrics.

d QoL: quality of life.

e PedsQL: Pediatric Quality of Life Inventory.

f EORTC PATSAT-C33: European Organisation for Research and Treatment of Cancer Satisfaction with Cancer Care Core Questionnaire.

g IOFS: Impact on Family Scale.

h HCP: health care professional.

Per protocol, the recruitment rate was defined as the proportion of recruited patients among eligible patients. The participation rate referred to recruited participants completing at least 70% of the 28 monthly questionnaires (before imputation). The attrition rate included patients who did not complete the 6-month study due to withdrawal or death. Reasons for missed assessments (multiple-choice and free-text responses) were documented when available.

Adherence was assessed by user group. For patients, adherence included the number of optional monthly symptom reports, diary entries, and in-game personalization activities. For parents, adherence included the number of monthly optional proxy symptom reports. For HCPs, adherence included the number of monthly treatment plans, reaction protocols, and proxy symptom reports. Engagement was defined as the frequency and consistency of platform use over time. Adherence referred to the completion of scheduled questionnaires, while participation described overall involvement in study activities.

Scoring and missing data handling followed the respective questionnaire guidelines (refer to Table 2). Additional details, including the full questionnaires, interview guides, coding schemes, paraphrased statements, and extended statistical outputs, are provided in Multimedia Appendix 1.

### Follow-Up Focus Groups

Focus groups were conducted at all three sites with (1) children and adolescents with cancer, their parents and (2) HCPs to obtain qualitative feedback on the platform (eg, experiences, challenges, barriers, and facilitators).

Semistructured interview guides, based on the mHealth App Usability Questionnaire [65], included prioritized, neutral, open-ended, unambiguous questions (refer to S3 in Multimedia Appendix 1 for patient and parent focus groups and S4 in Multimedia Appendix 1 for focus groups with HCPs). Patients and parents were approached post study. HCPs were divided into two subgroups: (1) project-internal HCPs who provided feedback during the platform’s development and (2) project-external HCPs who were involved only in the study.

Written informed consent was obtained before interviews (conducted in person or online and in the local language). Sessions lasted 45‐60 minutes and were moderated by trained local researchers. Audio recordings were transcribed verbatim, pseudonymized, and translated into English for analysis.

### Data and Statistical Analysis

Statistical analyses were conducted in R (version 4.3.2, R Core Team, R Foundation for Statistical Computing) [66]. Descriptive statistics summarized recruitment and baseline characteristics (frequency, mean [SD], or median [IQR], as appropriate). Tests were chosen based on variable distribution. Two-sided *P* values <.05 were considered significant.

Missing data were imputed according to questionnaire guidelines. For symptom reports, the highest monthly symptom burden per patient was considered. Missing responses (≤2 per measure) were replaced by the nearest previous value. The target sample size was 100 participants [45]. To accommodate missing data and the small sample size, repeated measures (baseline and M1-M6) were grouped into 4 time points (baseline, M1-M2, M3-M4, and M5-M6), ensuring no significant differences between merged time points via the two 1-sided tests, with odds ratios calculated as effect sizes.

The aligned rank transformation (ARTool; developed by Kay et al. [67]) enabled a nonparametric 2-factor repeated measures ANOVA for the factors of time, group, and their interaction effects [68]. Participants were grouped by (1) birth sex (female or male), (2) age (6-9, 10-13, and 14-17 years), (3) diagnosis (leukemia or solid tumor), and (4) Karnofsky Performance Status (KPS) [69,70] scale (below or above the cohort median). Partial eta squared (*η*_p_^2^) was classified as small (*η*_p_^2^≥0.01), medium (*η*_p_^2^≥0.06), and large effect size (*η*_p_^2^≥0.16). Post hoc comparisons used Wilcoxon signed-rank tests (time points) and Mann-Whitney *U* tests (group comparisons), both with Holm-Bonferroni correction. For significant interactions, rank-transformed time-point differences between groups were compared using Mann-Whitney *U* tests with Holm-Bonferroni correction.

Spearman rank correlation (*ρ*) assessed monotonic associations between patient self-reports and parent proxy reports of symptom burden per month (no imputation). Effect sizes were classified as weak (|ρ|≥0.1), moderate (|ρ|≥0.3), and strong (|ρ|≥0.5).

Qualitative focus group data were analyzed in MAXQDA 2022 (VERBI Software) using deductive and inductive approaches [71]. Two researchers independently reviewed transcripts and developed a consensus coding system for thematic analysis. Statements were cross-coded when applicable (eg, positive, neutral, and negative) and paraphrased after joint review.

Missing data handling, subgroup analyses, and model specifications were performed as described in this section. No formal adjustment for site-level differences was performed because of the limited sample size at each site.

### Ethical Considerations

The MyPal4Kids study protocol has obtained ethical approval from the ethics committee competent for the Saarland University (Ärztekammer des Saarlandes; reference: Ha 23/20; March 16, 2020), the ethics committee of the Hannover Medical School (reference: 9095_BO_K_2020; February 12, 2020), and the ethics committee of the University of Brno (reference: 01‐1 20 220/EK; May 11, 2020). This study was performed in line with the principles of the Declaration of Helsinki. Ethical and General Data Protection Regulation (GDPR) compliance considerations ensured data safety for this vulnerable participant group [48], as reflected in the protocol through age-appropriate information sheets and informed consent forms [45]. Informed consent was obtained from all participants and from parents or legal guardians of children or adolescents.

## Results

### Recruitment

**Figure 1.**
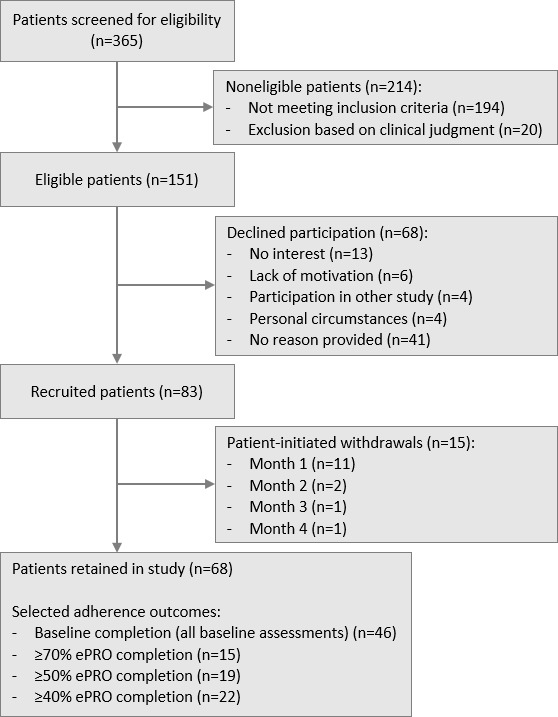
Flow diagram on screening, eligibility, and recruitment in MyPal4Kids. ePRO: electronic patient-reported outcome.

Recruitment took place over 15 months (December 2020-March 2022), including a 6-month COVID-19 extension. A total of 83 participants were enrolled (refer to Figure 1).

### Enrollment Characteristics

Table 3 summarizes the characteristics of the enrolled patients. Birth sex distribution and treatment status were nearly balanced. Almost half of the patients were aged 14‐17 years. Solid tumors accounted for nearly half of the cases, with brain tumors listed separately due to their potential neurological effects [72]. Most patients were newly diagnosed (70/83, 84%) and 16% (13/83) of patients had relapsed.

**Table 3.** Patient information collected at baseline.

Baseline characteristic	Study cohort (N=83), n (%)
Birth sex	
Male	46 (55)
Female	37 (45)
Age (years)	
6‐9	18 (22)
10‐13	25 (30)
14‐17	40 (48)
Diagnosis	
Brain tumor	14 (17)
Leukemia	29 (35)
Solid tumor	40 (48)
Stage	
New diagnosis	70 (84)
Relapse	13 (16)
On treatment	
Yes	45 (54)
No	38 (46)
KPS^a^	
Documented^b^^,^^c^	55 (66)
Not documented^d^	28 (34)

a KPS: Karnofsky Performance Status.

b Median (IQR): 80 (80-90).

c Range (min-max): 50-100.

d Nonmandatory information.

### Acceptability and Engagement With the MyPal Platform

#### Attrition, Participation, and Adherence Patterns

The attrition rate was 18% (15/83), with 73% of withdrawals at participants’ request (11/15) occurring in the first month (M1). Withdrawn participants included 8 male and 7 female participants; 8 had solid tumors, 6 had leukemia, and 1 had a brain tumor. The age distribution among those who discontinued participation was 10 aged 14‐17 years, 4 aged 10‐13 years, and 1 aged 6‐9 years. Most were newly diagnosed (n*=*14) and receiving treatment (n*=*9). Reasons for withdrawal (documented in 7 cases; multiple choice) included disease progression (n*=*4) and/or patient reluctance (n*=*6). No deaths occurred during participation.

Per protocol, 15 of 83 (18%) participants completed ≥70% of all monthly questionnaires, 19 (23%) completed ≥50%, and 22 (27%) completed ≥40%. Overall, 46 (55%) participants completed all baseline assessments (refer to Figure 1). Because these adherence thresholds were cumulative, the categories were not mutually exclusive. HCPs documented reasons for missed assessments in 32 cases (39%): patient reluctance (n*=*17) and disease progression (n*=*15).

**Figure 2.**
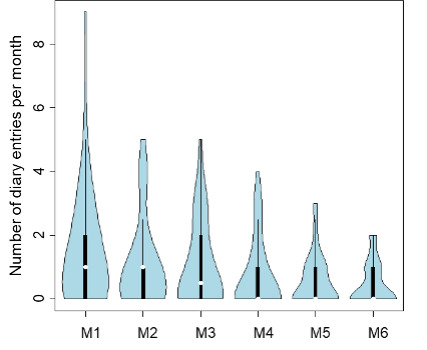
Violin plot depicting monthly frequencies of diary entries created by patients with at least one diary entry (n=42, 51%). In all violin plots, the width of the violin reflects the frequency of the respective measure, with white dots representing the median values, thick black bars indicating the IQR, and thin black bars indicating 1.5 x the IQR for each measure. Each violin represents the distribution across the entire study cohort for the respective month.

Of 83 participants, 62 (75%) patients assessed each symptom at least once. Assessment frequency declined over time. Overall, 42 (51%) patients created at least one diary entry, with the frequency declining over time (refer to Figure 2). In total, 64 (77%) patients customized their game avatar.

Regarding proxy reports on symptom burden, at least 53 (64%) parents assessed each symptom at least once. HCPs created overall 98 treatment plans for 29 (35%) patients, ranging from 1 to 8 plans per patient. HCPs documented symptoms through the HCP interface 15 times for 13 (16%) patients. HCPs added reactions to reported symptoms 24 times for 15 (18%) patients.

#### Usability of the MyPal Platform

Sixty of 83 patients and parents (72%) completed the respective version of the System Usability Scale (SUS). Among responding patients, 30% (18/60) were younger than 10 years, whereas 70% (42/60) were 10 years or older. Younger children largely found the game enjoyable and easy, whereas older patients reported more variable ratings (refer to Table 4). A significant association with a small effect size was found between age group and game enjoyment (Q1; *χ*^2^_1_=4.4; *P*=.04; *φ*=−0.27).

**Table 4.** Answers to the adapted SUS^a^ evaluating the usability of the MyPal-Child app were received from 60 of 83 patients.

Questionnaire items	Yes, n (%)	No, n (%)	Indecisive, n (%)
Adapted SUS^a^ for children <10 years (n*=*18)			
Q1: Did you like the game?	15 (83)	3 (17)	0 (0)
Q2: Was the game easy to play?	15 (83)	2 (11)	1 (6)
Q3: Did you like to answer questions in the game?	10 (56)	8 (44)	0 (0)
Adapted SUS for children≥10 years (n*=*42)			
Q1: Did you like the game?	23 (55)	19 (45)	0 (0)
Q2: Was the game easy to play?	39 (93)	2 (5)	1 (2)
Q3: Did you like to answer questions in the game?	21 (50)	21 (50)	0 (0)
Q4: Were the questions asked to you relevant at the time?	25 (60)	15 (36)	2 (5)
Q5: Was the frequency of the reminders to answer questions appropriate?	24 (57)	18 (43)	0 (0)

a SUS: System Usability Scale.

Free-text comments from 34 (57%) patients mentioned avatar customization, points collection, and graphics as enjoyable features. Some found the game too simple and repetitive and suggested increased difficulty, customization options, and additional achievements. Parents rated the MyPal-Carer app highly for usability (mean SUS 76.8, SD 15.9; median 82.5, IQR 63.1‐90), indicating good to excellent usability [55].

### Feasibility of ePRO-Based Measures

#### Patient and Proxy-Reported Symptom Burden

**Figure 3.**
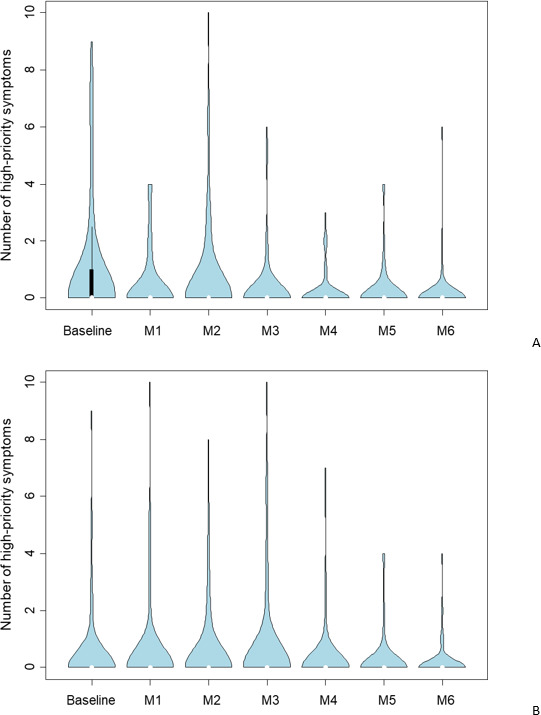
Frequency distribution of monthly symptom reports with high priority (ie, the highest burden) via Symptom Screening in Pediatrics or Mini Symptom Screening in Pediatrics during study participation. Each violin represents the distribution across the entire study cohort for the respective month. (**A**) Self-reports by children and (**B**) proxy reports by parents for their children.

Overall, 42 of 83 (51%) participants reported at least one symptom with the highest burden (rating 4=extremely bothered) during the 6-month period (Figure 3A). The most frequent symptoms were tiredness (29 reports), pain (other than headache; 26 reports), mouth sores (23 reports), and changes of appetite (23 reports). Less than 50% of patients reported at least one symptom with the highest burden per month.

Overall, 34 of 83 (41%) parents submitted proxy reports (refer to Figure 3B), citing feeling disappointed or sad (24 reports), perceived changes in appearance (23 reports), and changes in appetite (23 reports) with the highest burden. Thus, some overlap existed between child and parent reports.

Spearman rank correlation across 15 symptoms over 7 time points revealed significant correlations for 8 symptoms at up to 3 time points each (refer to Table 5), ranging from ρ=0.39 to ρ=1.0 (moderate to strong), but inconsistently across time.

**Table 5.** Spearman rank correlation ρ between children’s and parents’ reported symptom burden via Symptom Screening in Pediatrics or Mini Symptom Screening in Pediatrics across time points.

Symptom and time point	df (n - 2)	ρ
Feeling disappointed or sad		
Baseline	24	0.18
M1	22	0.09
M2	22	0.16
M3	13	0.21
M4	13	0.14
M5	12	0.62^a^
M6	6	0.51
Feeling scared or worried		
Baseline	25	0.22
M1	21	0.48^a^
M2	22	0.46^a^
M3	12	−0.16
M4	13	0.11
M5	13	0.45
M6	6	0.59
Feeling cranky or angry		
Baseline	24	0.39^a^
M1	22	0.58^b^
M2	21	0.54^b^
M3	11	0.19
M4	14	0.16
M5	12	0.46
M6	5	0.73
Changes in how body or face look		
Baseline	22	0.14
M1	21	0.39
M2	22	0.10
M3	12	0.28
M4	14	−0.11
M5	12	0.57^a^
M6	5	0.87^a^
Feeling tired		
Baseline	21	0.22
M1	21	0.67^c^
M2	22	0.49^a^
M3	11	0.52
M4	14	0.30
M5	12	0.20
M6	4	0.67
Feeling pain		
Baseline	19	0.18
M1	20	0.47^a^
M2	21	0.26
M3	12	0.38
M4	13	0.36
M5	11	0.42
M6	4	1.0^c^
Tingly or numb hands or feet		
Baseline	19	0.20
M1	20	0.62^b^
M2	21	0.54^b^
M3	11	0.32
M4	13	0.31
M5	11	0.22
M6	4	0.50
Vomiting or nausea		
Baseline	18	0.30
M1	20	0.39
M2	20	0.28
M3	11	0.40
M4	14	−0.30
M5	11	0.71^b^
M6	4	0.72

a *P*<.05.

b *P*<.01.

c *P*<.001.

#### Subgroup and Longitudinal Differences in Symptom Burden and QoL

**Figure 4.**
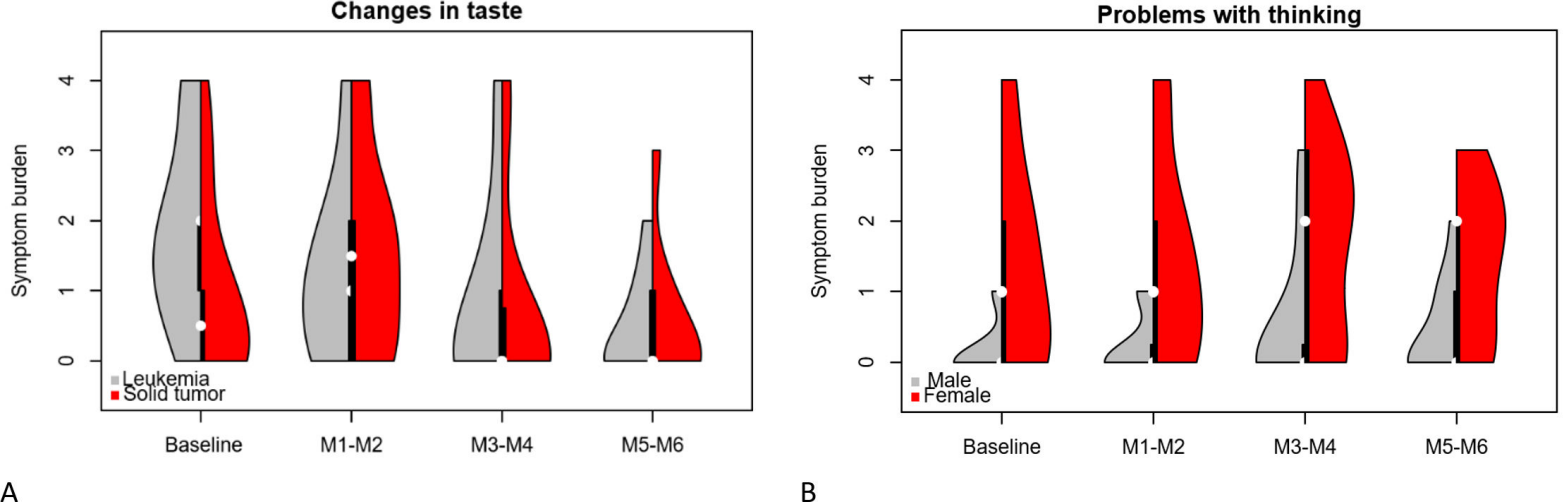
Violin plots depicting the distribution of maximum burden (ranging from 0=not at all bothered to 4=extremely bothered) for 2 Symptom Screening in Pediatrics or Mini Symptom Screening in Pediatrics symptoms across 4 timepoints from baseline until the final 2 months of study participation (M5-M6).

Table 6 summarizes results from the aligned rank transformation analysis, excluding symptoms without significant findings. KPS-based subgroups were categorized as above or below the median score of 90 (IQR 80‐90). For example, a significant main effect of time was found for changes in taste (Figure 4A), with symptom burden decreasing from M1-M2 to M5-M6 regardless of group according to post hoc comparisons (birth sex: *P=*.003*, η*_p_^2^*=*0.151; age: *P=*.01*, η*_p_^2^*=*0.124; diagnosis: *P=*.007*, η*_p_^2^*=*0.118; KPS: *P=*.02*, η*_p_^2^*=*0.128). Regarding problems with thinking, a significant main effect of group was found, with symptom burden being higher among females than among males, regardless of time (Figure 4B).

**Table 6.** Symptoms reported via Symptom Screening in Pediatrics or Mini Symptom Screening in Pediatrics with significant results derived from the aligned rank transformation analysis.

Symptoms, groups, and subsamples	Imputed, n	Factor time		Factor group		Interaction time × group	
		*P* value	𝜂_p_^2^	*P* value	𝜂_p_^2^	*P* value	𝜂_p_^2^
Feeling tired							
Birth sex	13	0.13	0.069	0.03	0.167^a^	0.74	0.016
Female (n=16)							
Male (n=12)							
More or less hungry than usual (changes in appetite)							
Birth sex	12	0.73	0.017	0.02	0.194^a^	0.74	0.019
Female (n=16)							
Male (n=11)							
Mouth sores							
Diagnosis	12	0.04	0.102^b^	0.02	0.190^a^	0.86	0.01
Leukemia (n=9)							
Solid tumor (n=18)							
Headache							
KPS^c^	10	0.004	0.183^a^	0.85	0.002	0.1	0.09
KPS ≥80 (n=11)							
KPS <80 (n=13)							
Tingly or numb hands/feet							
Diagnosis	12	0.01	0.136^b^	0.36	0.033	0.31	0.046
Leukemia (n=9)							
Solid tumor (n=18)							
Changes in how body or face look							
Birth sex	13	0.02	0.109^b^	0.29	0.041	0.41	0.035
Female (n=17)							
Male (n=12)							
Age group (y)	13	0.01	0.134^b^	0.85	0.012	0.99	0.009
6‐9 (n=11)							
10‐13 (n=8)							
14‐17 (n=10)							
Diagnosis	13	0.003	0.160^a^	0.5	0.017	0.32	0.042
Leukemia (n=9)							
Solid tumor (n=20)							
KPS	9	0.02	0.144^a^	0.81	0.003	0.41	0.042
KPS ≥80 (n=11)							
KPS <80 (n=13)							
Changes in taste							
Birth sex	12	<.001	0.248^a^	0.34	0.037	0.69	0.019
Female (n=16)							
Male (n=11)							
Age group (y)	12	0.001	0.198^a^	0.87	0.011	0.83	0.038
6‐9 (n=10)							
10‐13 (n=8)							
14‐17 (n=9)							
Diagnosis	12	<.001	0.284^a^	0.56	0.014	0.02	0.123^b^
Leukemia (n=9)							
Solid tumor (n=18)							
KPS	10	0.001	0.223^a^	0.54	0.017	0.7	0.021
KPS ≥80 (n=11)							
KPS <80 (n=13)							
Vomiting or nausea							
Birth sex	12	0.009	0.142^a^	0.47	0.021	0.18	0.063
Female (n=16)							
Male (n=11)							
Age group (y)	12	0.003	0.175^a^	0.76	0.021	0.04	0.161^a^
6‐9 (n=10)							
10‐13 (n=8)							
14‐17 (n=9)							
Diagnosis	12	0.005	0.157^a^	0.91	0.001	0.81	0.013
Leukemia (n=9)							
Solid tumor (n=18)							
Problems with thinking and remembering things							
Birth sex	13	0.57	0.024	0.001	0.321^a^	0.56	0.025
Female (n=17)							
Male (n=12)							
KPS	9	0.95	0.005	0.15	0.092	0.03	0.123^b^
KPS ≥80 (n=11)							
KPS <80 (n=13)							
Diarrhea							
Birth sex	12	0.08	0.084	0.16	0.076	0.03	0.108^b^
Female (n=16)							
Male (n=11)							
Age group (y)	12	0.17	0.066	0.3	0.096	0.02	0.181^a^
6‐9 (n=10)							
10‐13 (n=8)							
14‐17 (n=9)							
Diagnosis	12	0.002	0.181^a^	0.27	0.048	0.009	0.143^a^
Leukemia (n=9)							
Solid tumor (n=18)							
Constipation							
Birth sex	12	0.14	0.07	0.3	0.043	0.03	0.111^b^
Female (n=17)							
Male (n=12)							

a Large effect size (*η*_p_2).

b Medium effect size (*η*_p_2).

c KPS: Karnofsky Performance Status.

**Figure 5.**
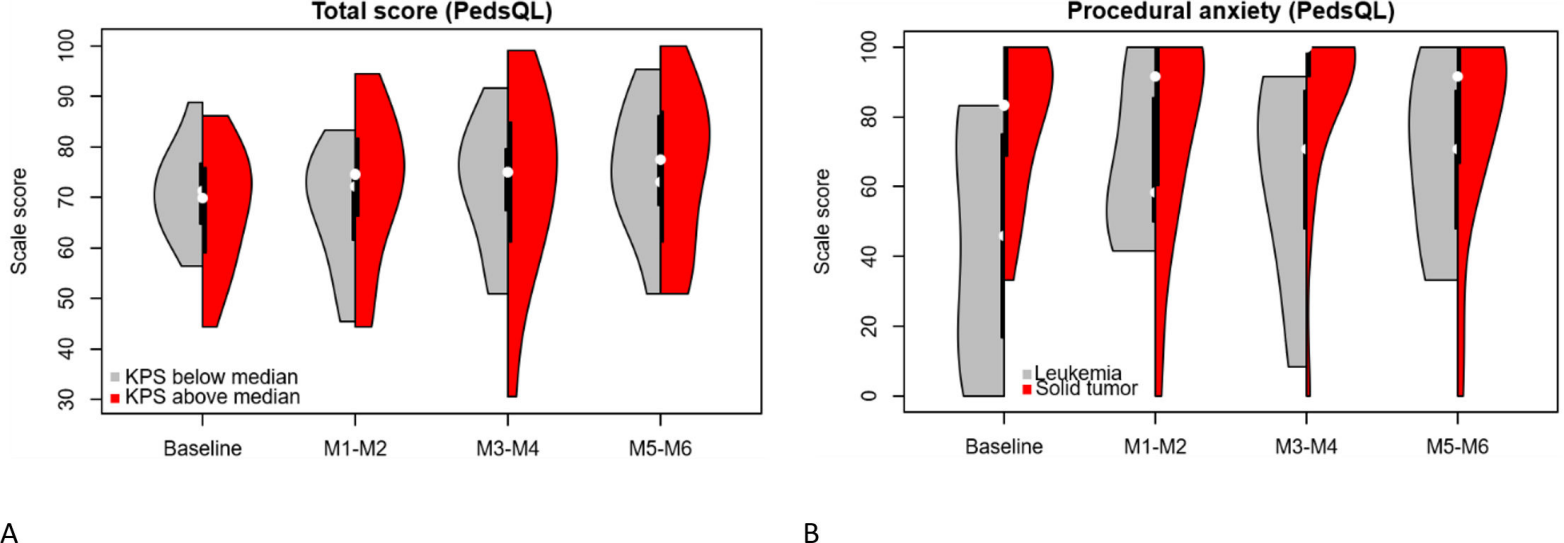
Violin plots depicting the distribution of 2 PedsQL dimensional scores, reported by the respective subgroups across 4 timepoints from baseline until the final 2 months of study participation (M5-M6). KPS: Karnofsky Performance Status; PedsQL: Pediatric Quality of Life Inventory.

Similarly, time, group, and interaction effects emerged for the other ePRO measures. For example, there was a significant main effect of time for the children’s QoL total score regardless of group. Post hoc tests revealed a significant increase from baseline to M5-M6 (*P=*.02; *η*_p_^2^*=*0.126; Figure 5A). A significant main effect of group (Figure 5B) was found for procedural anxiety, regardless of time: problems with procedural anxiety were more prevalent in patients with leukemia and in the youngest age group compared with patients with solid tumors and the oldest age group (*P=*.009; *η*_p_^2^*=*0.285). Full statistical results for these ePRO measures (Pediatric Quality of Life Inventory [PedsQL], European Organisation for Research and Treatment of Cancer Satisfaction with Cancer Care Core Questionnaire [PATSAT-C33], and Impact on Family Scale [IOFS]), including subgroup analyses, are provided in S6 in Multimedia Appendix 1.

#### Focus Groups With Children and Their Parents

Ten parent-child pairs participated: 3 at USAAR, 4 at MHH, and 3 at FNBRNO, across all age groups: 2 aged 6‐9 years, 2 aged 10‐13 years, and 6 aged 14‐17 years. The coding system linking 18 categories to statements and their frequencies is shown in S7 in Multimedia Appendix 1. Most comments concerned the categories “Recommendations,” followed by “Game Design” and “ePROs.” Key findings are provided in Table 7, while the full coding scheme, category frequencies, and paraphrased statements are provided in S7 in Multimedia Appendix 1.

**Table 7.** Key findings from the focus groups with children and their parents.

Code category	Summary
Game Design	Children frequently mentioned enjoying the customization features and underwater theme. Comments on gameplay included perceptions of repetitiveness and limited challenge.
ePROs^a^	Parents appreciated symptom reporting but noted some children underreported, often due to fatigue. Both parents and children found the questions understandable, but some described the questions as repetitive or less relevant during symptom-free periods.
Consequences of App Usage	The app felt most beneficial at the treatment’s onset. However, engagement decreased over time. It was useful for self-reflection and documenting symptoms, particularly when direct communication with HCPs^b^ was limited.
Communication	The app was not commonly perceived as improving communication but was reported as enabling some children to express feelings outside face-to-face interactions. However, infrequent feedback from HCPs in response to symptom reports led to frustration.
Recommendations	Participants suggested expanding gameplay variety, customization options, tailoring the App for different age groups, and adding multiplayer modes and peer support as social interaction features, as well as improving HCP feedback to enhance engagement.

a ePRO: electronic patient-reported outcome.

b HCP: health care professional.

### Impact on HCPs Due to the Integration of ePROs in Care

#### Follow-Up Questionnaire for HCPs

**Figure 6.**
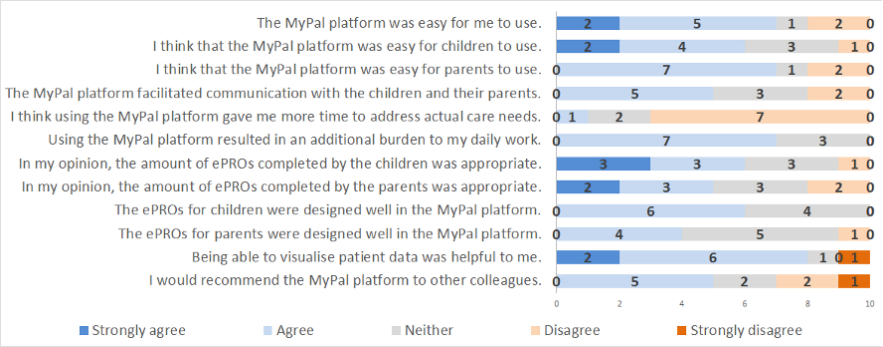
Distribution of the absolute frequencies of the health care professionals’ responses to the items of the web-based follow-up questionnaire (n=10). ePRO: electronic patient-reported outcome.

Ten HCPs (5 project-internal and 5 project-external) completed the follow-up questionnaire (refer to Figure 6). No significant group differences were found. Half added free-text comments, praising online communication when in-person appointments were not possible. They valued ePROs and the idea of the platform but stressed the importance of personal contact. Concerns included missing data from disengaged participants. Recommendations included better integration with existing clinical systems, enhanced data visualization, and improved game design.

#### Focus Groups With HCPs

Nine HCPs joined focus groups: 4 physicians (2 from MHH, 1 from FNBRNO, and 1 from USAAR), 1 nurse (MHH), and 1 psychologist (MHH) as project-internal, along with 1 physician (MHH), 1 psychologist (MHH), and 1 physician (FNBRNO) as project-external. The coding mirrored parent-child pair focus groups, but “Game Design” and “ePROs” were addressed less, while “Influences on Motivation,” “HCP-Patient Communication,” and “Compliance” had higher coverage. Compared to internal HCPs, external HCPs provided fewer statements coded as negative during focus group discussions and discussed user experiences and suggestions more than internal HCPs. Tables 8 and 9 summarize the internal and external HCP focus groups, respectively. The full coding schemes, category frequencies, and paraphrased statements are provided in S7 in Multimedia Appendix 1.

**Table 8.** Key findings from the focus groups with project-internal HCPs^a^.

Code category	Summary
Game Design	HCPs^a^ observed that older children were less engaged, mentioning limited variety and a perceived lack of competitiveness in the game.
Expectations	Initial expectations ranged from mixed curiosity to skepticism regarding the platform’s ability to sustain engagement and seamlessly integrate into clinical workflows.
Consequences of App Usage	HCPs noted that some patients seemed to avoid the app as it reminded them of their illness, leading to disengagement, especially at home.
Communication	The app served as an additional communication channel, particularly for teenagers who preferred expressing themselves digitally. However, its impact on team communication seemed to highly depend on the strength of existing communication between HCPs, patients, and families. In cases of weaker communication, digital tools were seen as helpful for capturing and preserving important information.
Influences on Motivation	Initial curiosity waned due to limited feedback. HCPs reported more consistent use among girls and follow-up patients compared to boys or those in acute care.
Recommendations	HCPs recommended incorporating automated data collection (eg, wearables) and enhancing the HCP interface for better notification management. However, they also highlighted the need to address data protection and institutional barriers in general before effective implementation is possible.

a HCPs: health care professionals.

**Table 9.** Key findings from the focus groups with project-external HCPs^a^.

Code category	Summary
Game Design	External HCPs^a^ found the game’s long-term engagement insufficient, citing competition with highly stimulating commercial games as a major hurdle.
Expectations	Initially, HCPs were optimistic about the app providing a constructive, meaningful alternative to passive media consumption for children.
Consequences of App Usage	HCPs described limited changes in daily workflows, although some noted occasional use for symptom tracking. Families sometimes used it to recall symptoms during appointments, but it did not seem to substantially alter HCP working routines.
Communication	The app aided communication for children more comfortable with digital tools, though HCPs emphasized that digital reporting should complement, not replace, in-person interactions.
Influences on Motivation	HCPs observed reduced engagement over time, particularly as patients’ health improved. Parents of younger children were more engaged in symptom tracking, while older children primarily used the diary function for information sharing. Participants described engagement as influenced by usage habits, perceived utility, and occasional technical challenges (eg, login issues).
Impression of eHealth^b^ Solutions	HCPs were generally optimistic about the growing role of eHealth but stressed that digital tools must complement traditional care. Challenges included administrative barriers and insufficient digital infrastructure.
Recommendations	External HCPs suggested additional personalized game features to increase long-term engagement and enhance the app interface for HCPs, including a priority system to highlight urgent issues.

a HCPs: health care professionals.

b eHealth: electronic health solution.

## Discussion

### Principal Findings

MyPal4Kids assessed the feasibility and acceptability of an ePRO-based platform in pediatric oncology and palliative care. Patient engagement was strongly influenced by perceived system utility, linked to HCP feedback frequency and the game’s ability to sustain motivation. The core hypothesis that gamification would make self-reporting more engaging for young patients was partially supported: initial interest was high, but long-term engagement declined. These findings align with research emphasizing bidirectional communication in digital health [73-75], while also highlighting implementation challenges.

A key challenge was a disengagement cycle: fewer patient reports led to fewer platform reviews by HCPs, reducing feedback and further discouraging engagement. Similar patterns have been observed in other studies [76], likely amplified by the emotional burden on young patients with cancer and their families, along with competing HCP priorities. Usability did not limit patient engagement in our study but was more relevant for HCP adoption. These findings suggest that all groups may require additional support, such as proactive feedback loops and tighter integration into clinical workflows and systems, to maintain engagement. Engagement decreased over time, which represents an important finding of this study. This pattern was analyzed using descriptive longitudinal summaries rather than formal subgroup modeling. Engagement reflects a behavioral usage process characterized by heterogeneous exposure durations, irregular participation intervals, and varying initiation and discontinuation points across participants. This differs from structured repeated-measures clinical outcome assessments collected at predefined time points, which are more suitable for subgroup comparisons within the feasibility framework. To complement the quantitative description, we also further provide stratified descriptive observations (eg, age-related differences in usability and engagement patterns) alongside qualitative findings that help contextualize these trends, including differences in motivation, disease status, and HCP feedback.

Potential explanations for the observed decline include limited novelty effects, insufficient personalization of gamification elements, and varying needs across age groups. Additionally, the degree of feedback from HCPs may have influenced sustained use. These findings highlight the need for adaptive and user-centered design strategies to maintain long-term engagement. In addition to the COVID-19 pandemic, institutional factors such as varying levels of digital infrastructure, staffing resources, and staff engagement may have influenced implementation processes and platform use across study sites.

Similar engagement trajectories have been reported in pediatric digital health and ePRO-based interventions, where engagement is typically high at initiation but declines over time despite generally acceptable usability. Prior pediatric ePRO studies have similarly described decreases in reporting frequency in longitudinal implementations [77-79], as well as in broader mHealth and game-based interventions [80,81]. These findings are consistent with evidence that gamification strategies often produce short-term engagement effects but are insufficient to sustain long-term use [30]. In addition, the effectiveness of social or competitive design elements may depend on a sufficiently large and continuously active user base, which is often lacking in pediatric feasibility settings [82]. Together, these observations suggest that sustained engagement requires more than initial usability and gamification and depends on continued perceived relevance, feedback mechanisms, and integration into clinical workflows.

### Platform Utility for Clinical Care and Research

Importantly, this study was designed as a feasibility study and was not intended to evaluate the effectiveness of the intervention. Therefore, any observed changes in ePROs should be interpreted descriptively and not as evidence of causal effects. The platform proved feasible for tracking individual symptom burden over time, contingent on patient engagement in reporting symptoms. Clinicians could benefit from reviewing individual symptom trajectories. Statistical analyses showed significant main and interaction effects of time and group. Larger datasets could enable more nuanced comparisons, enhancing the platform’s potential for research applications, such as comparing treatments in terms of effectiveness or side effects. This dual capability, supporting individualized patient care while generating population-level insights, underscores the platform’s potential for both clinical practice and research. While symptom burden was the primary focus, secondary measures like children’s QoL also showed improvement from baseline until the end of the study. Interpretation of these findings should remain cautious, as broader pandemic-related disruptions may have influenced reporting behaviors, engagement patterns, and ePROs during the study period [83-85].

### Contextual Factors Influencing Implementation

Underlying factors may include age, digital literacy, emotional strain, and institutional support for digital health tools. Assessed symptoms largely reflected expected chemotherapy side effects, about which patients and parents had prior information. Consequently, HCPs sometimes gave limited direct feedback. Still, reports of symptoms such as tingling or numbness in the extremities are clinically relevant, potentially guiding treatment adjustments of medication doses. Our findings reinforce concerns that insufficiently integrated digital health tools can increase rather than reduce workload for HCPs, particularly when clear integration pathways into routine clinical workflows and responsibilities are lacking [75,86,87]. In the present study, the platform was deliberately not embedded into mandatory clinical workflows, which may have contributed to this effect. Existing commercial clinical systems offer limited interoperability with digital health platforms, including electronic health record integration, application programming interfaces and authentication infrastructures (eg, different user accounts and password systems). This complicates seamless integration into routine clinical workflows and requires the use of parallel systems. Dedicated staff to review digital health data could alleviate this burden, an approach supported in the literature [12]. Timely symptom data are crucial for responsive care, particularly between infrequent visits [88]. The observed decline in symptom reporting mirrors similar studies, possibly due to symptom relief or engagement fatigue. While gamification sparked initial interest, it alone was insufficient to sustain engagement, especially in older children. The game’s design was constrained by requirements for nonviolence, gender neutrality, and broad age group appeal. While these specifications ensured accessibility, they may have resulted in an overly cautious design. Concerns about overuse did not materialize. Established gamification elements such as rewards and competitive features were less effective without a critical mass of simultaneous active users. Perspective differences between internal and external HCPs should be considered. Internal HCPs, involved in platform development, focused on long-term potential, while external HCPs emphasized practical challenges like workflow integration. Both groups agreed that technology should complement, not replace, direct care [89].

### Strengths and Limitations

Interdisciplinary collaboration and testing across multiple clinical sites strengthened the study. Patient, parent, and HCP involvement in the design and evaluation enhanced the relevance of the findings. Ethical considerations were central to platform development and study implementation. Integration of ePROs into a gamified mobile app for young patients with cancer was innovative, aligning with evidence that children prefer visually engaging digital measures with short recall periods [90]. A strength of the presented platform is its flexibility for various ePROs and gamification strategies, enabling implementation across varied contexts.

The study period overlapped with the COVID-19 pandemic, which likely affected recruitment, clinical workflows, and sustained platform engagement. Reduced face-to-face interactions, restrictions during clinical visits, and increased reliance on digital communication may have influenced onboarding, participant retention, and reporting behavior. Prolonged exposure to digital communication and online platforms during the pandemic may also have contributed to digital fatigue and declining engagement over time. Similar pandemic-related disruptions affecting recruitment, ePRO-based research, and health-related QoL assessments have been described in pediatric and digital health research [83,85,91].

Further limitations include discrepancies between child and parent symptom reports, possibly due to recall issues or differing perceptions. While proxy reports can increase data completeness, they may introduce bias, highlighting the importance of careful interpretation and, where possible, cross-checking [92]. Some parents may have prioritized immediate care over proxy reports. Dual reporting was optional, limiting analyses to overlapping cases. Still, at certain time points, some symptoms demonstrated strong parent-child correlations, supporting limited proxy validity. MyPal was not designed as an emergency tool, as stated in the consent. Legal and ethical concerns, such as liability for missed reports, implicated that HCPs were not expected to monitor reports constantly. This limited the platform’s immediate clinical utility but matched its intended use as a supplemental communication channel. The small sample size limits the robustness and generalizability of the findings. Given the limited sample size and reduced numbers in subgroup analyses, these findings should be interpreted with caution. The results are exploratory and intended to inform future studies rather than provide definitive conclusions. The 6-month study period may not reflect long-term patterns. Given the drop in engagement, longer studies may require design refinements. The sample was limited in terms of diversity (eg, age and disease types). Selection bias is possible, as families with higher digital literacy or strong clinical engagement may have been more likely to participate. The overrepresentation of adolescents (aged 14‐17 years) does not reflect the general pediatric cancer population [93]. Younger children younger than 4 years have the highest cancer incidence but were excluded due to literacy barriers. Treatment plan creation in the platform was optional for HCPs, limiting analysis of symptom-treatment correlations. Recruitment and HCP availability were likely affected by the pandemic, though the impacts are hard to quantify. Emotional burden during early illness phases also influenced recruitment. Subjective feedback from participants within the clinical setting may have shaped both recruitment and engagement. Measures such as flexible baseline and monthly assessment windows, as well as offline functionality, were implemented to support compliance, though their success was limited. Secondary end point measures (eg, patients’ and parents’ QoL, care satisfaction, and family impact) were not available to HCPs during the study. Access to these data could increase HCP engagement.

Taken together, these factors may limit the generalizability of the findings beyond European pediatric oncology settings, particularly in contexts with different health care infrastructures, cultural expectations, and levels of digital literacy.

### Implications and Future Directions

Although more digital health solutions are emerging for specific illness phases, creating comprehensive platforms that address diverse individual needs remains challenging. Concerns persist about using research-oriented ePRO measures in clinical decision-making [8]. Pediatric-specific clinical ePRO guidelines are lacking [94], underscoring the need for evidence to support standardization and broader clinical adoption. Future versions should consider structured HCP training and targeted family support. Embedding ePROs into standard care rather than offering them as optional tools may help sustain use. Refinements should reflect user feedback and improve age-specific adaptability to enable scalability across clinical settings. Hybrid models, combining automated feedback with periodic HCP review, may boost engagement and long-term utility. Digital health solutions may not suit all patients; tailoring them to specific populations is key. Longitudinal randomized controlled trials are needed to assess effectiveness. Future research should explore the use of the platform in different patient populations, such as long-term survivors, who may demonstrate different supportive care needs and engagement patterns. In addition, studies conducted in postpandemic settings may help clarify the extent to which engagement patterns, recruitment dynamics, and sustained platform use were influenced by pandemic-related contextual factors. Evaluating the platform in larger cohorts and in more controlled study settings will be important next steps.

In conclusion, integrating digital health tools into pediatric oncology and palliative care requires both technological innovation and thoughtful workflow integration. Sustaining participation and utility demands consistent HCP engagement and compelling gamification. Large-scale implementation will require dedicated resources, including staff time for data monitoring, integration into existing hospital IT infrastructures and application programming interfaces, authentication systems, and sustainable allocation of clinical responsibility for digital symptom review. Our findings highlight key areas for refinement to improve ePRO-based approaches before clinical use is feasible.

## Supplementary material

10.2196/80718 Multimedia Appendix 1 Additional study materials, including questionnaires, focus group guides, detailed information on outcome measures, and extended quantitative and qualitative results.

## Abbreviations

EORTC PATSAT-C33: European Organisation for Research and Treatment of Cancer Satisfaction with Cancer Care Core Questionnaire
ePRO: electronic patient-reported outcome
FNBRNO: University Hospital Brno
GDPR: General Data Protection Regulation
HCP: health care professional
IOFS: Impact on Family Scale
IOFS: Impact on Family Scale
KPS: Karnofsky Performance Scale
MHH: Hannover Medical School
PedsQL: Pediatric Quality of Life Inventory
PedsQL: Pediatric Quality of Life Inventory
QoL: quality of life
SUS: System Usability Scale
USAAR: Saarland University
