# Supplementary material for: Feasibility of Implementing an Electronic Patient-Reported Outcome-Based Digital Health Platform in Pediatric Oncology and Palliative Care: Insights From the MyPal4Kids Multicenter Observational Study
Source: JMIR Cancer. 2026 Aug 11;12:e80718. doi: 10.2196/80718 (PMC13460066; doi:10.2196/80718)
Supplement: Supplementary file 1 [file cancer-v12-e80718-s001.pdf]

Supplement

Table of Content

Supplement 1

S1: System Usability Scale Adapted for Children 2

S2: Web-Based Questionnaire for HCPs 3

S3: Follow-up Focus Group With Patients and Their Parents 5

S4: Follow-up Focus Group With HCPs 6

S5: Additional Information on Secondary Endpoints And ePRO-Based Measures 7

Table S5: Overview of secondary endpoints and ePRO-based measures 7

S6: Extended Results On Feasibility Of ePRO-Based Measures 7

Table S6.1: PedsQL^{TM} dimensional scores with significant results derived from the aligned rank transformation analysis 8

Table S6.2: PATSAT scales with significant results derived from the aligned rank transformation analysis. 9

Table S6.3: IOFS factors with significant results derived from the aligned rank transformation analysis... 10

S7: Extended Results Of Focus Groups 11

S7.1: Focus Groups With Children And Parents 11

S7.2: Focus Groups With Project-Internal HCPs 14

S7.3: Focus Groups With Project-External HCPs 16

S1: System Usability Scale Adapted for Children

Version for children < 10 years

Dear AquaScout!

Thank you for taking part in the MyPal4Kids study!

Please answer the following questions about the App:

1. Did you like the game? Yes | No

2. Was the game easy to play? Yes | No

3. Did you enjoy answering questions in the game? Yes | No

Please write a few words about what you liked or disliked about the App and the game:

Free text answer

Version for children > 10 years

Dear AquaScout!

Thank you for taking part in the MyPal4Kids study!

Please answer the following questions about the App:

1. Did you like the game? Yes | No

2. Was the game easy to play? Yes | No

3. Did you enjoy answering questions in the game? Yes | No

4. Were the questions that were asked appropriate at the time? Yes | No

5. Was the frequency of reminders to answer questions appropriate? Yes | No

Please write a few words about what you liked or disliked about the App and the game:

Free text answer

S2: Web-based Questionnaire for HCPs

The impact on HCPs due to the integration of ePROs in palliative care

We are interested in understanding your experiences of using the MyPal platform. Below are a number of statements about the MyPal platform. Please indicate to what extent you agree or disagree with the following statements:

1) Were you already involved in the MyPal project before the study period?
    - Yes
    - No

2) The MyPal platform was easy for me to use.
    - Strongly agree
    - Agree
    - Neither agree nor disagree
    - Disagree
    - Strongly disagree

3) I think that the MyPal platform was easy for children to use.
    - Strongly agree
    - Agree
    - Neither agree nor disagree
    - Disagree
    - Strongly disagree

4) I think that the MyPal platform was easy for parents to use.
    - Strongly agree
    - Agree
    - Neither agree nor disagree
    - Disagree
    - Strongly disagree

5) The MyPal platform facilitated communication with the children and their parents.
    - Strongly disagree
    - Disagree
    - Neither agree nor disagree
    - Agree
    - Strongly agree

6) I think using the MyPal platform gave me more time to address actual care needs.
    - Strongly agree
    - Agree
    - Neither agree nor disagree
    - Disagree
    - Strongly disagree

7) Using the MyPal platform resulted in an additional burden to my daily work.
    - Strongly agree
    - Agree
    - Neither agree nor disagree
    - Disagree
    - Strongly disagree

8) In my opinion, the amount of ePROs completed by the children was appropriate.
    - Strongly agree
    - Agree
    - Neither agree nor disagree
    - Disagree
    - Strongly disagree

9) In my opinion, the amount of ePROs completed by the parents was appropriate.
    - Strongly agree
    - Agree
    - Neither agree nor disagree
    - Disagree
    - Strongly disagree

10) The ePROs for children were designed well in the MyPal platform.
    - Strongly agree
    - Agree
    - Neither agree nor disagree
    - Disagree
    - Strongly disagree

11) The ePROs for parents were designed well in the MyPal platform.
    - Strongly agree
    - Agree
    - Neither agree nor disagree
    - Disagree
    - Strongly disagree

12) Being able to visualize patient data was helpful to me
    - Strongly agree
    - Agree
    - Neither agree nor disagree
    - Disagree
    - Strongly disagree

13) I would recommend the MyPal platform to other colleagues
    - Strongly agree
    - Agree
    - Neither agree nor disagree
    - Disagree
    - Strongly disagree

14) Would you like to continue using the MyPal platform?
    - Yes
    - No

15) Please provide further comments / suggestions / remarks about the MyPal platform and its features in the following box: Freetext answer

S3: Follow-up Focus Group With Patients and Their Parents

1. If you had to explain to a friend in a few sentences your impression of the Apps, what would you say? **[children = c and parents = p]**
    a. What did you like most and what least about the Apps? **[c and p]**
2. How did you find the questions and answer options that popped up during the game? **[only c]**
    a. Would you change any of them? If yes, what would you change? **[only c]**
    b. What did you find helpful? **[only c]**
3. Talking about one’s state of health and mood often is not easy, sometimes even uncomfortable: How did you feel about frequently reporting about your mood and state of health? **[only c]**
    a. How much did the App help you with it? **[only c]**
    b. How would you describe the impact the App had for you, dealing with your condition and mood? **[only c]**
    c. Now that the study is over, can you identify any benefits or drawbacks for you or your child, of the App’s use?” **[only p]**
    d. Have you noticed any differences in the way your child communicates about his/her health? Would you say it improved or got worse? **[only p]**
4. What should have been done so that you used the Apps more often? **[c and p]**
    a. In your opinion, what extras should the Apps have? **[c and p]**
    b. What would you leave out or change? **[c and p]**
5. How much have the doctors and nurses responded to what you have shared through the App? **[only c]**
    a. What did you think of the frequency and the content when your physician referred to it? **[only c]**
    b. How did you wish it to be? **[only c]**
6. Imagine you are an inventor, and you have the chance to invent your own App that helps you, other children and their relatives with the illness, what will your App look like? What would your App be able to do? **[c and p]**
7. When we first introduced you to the App, you certainly had some thoughts or feelings towards it: What did you expect from the Apps? **[c and p]**
    a. Have your expectations been met and why (not)? **[c and p]**
    b. What are the Apps lacking according to your preferences? **[c and p]**
    c. How time consuming was it for you to use the Apps? **[c and p]**
8. Sometimes we do not feel like playing or answering questions: But when you did use the App, what made you use the App? What motivated you? **[only c]**
    a. What were the reasons why you did NOT use the App regularly? **[only c]**
9. You really helped us during the time of the project. Now the project is almost over. Could you imagine continuing to use the Apps? **[only c]**

S4: Follow-Up Focus Group With HCPs

1. When we first introduced you to the MyPal platform and the Apps, what were your thoughts or feelings towards it: What did you expect from it?
2. After using the MyPal platform and the Apps, please tell us about the following:
    a. What would you leave out or change?
    b. What is missing that you think should be included?
3. What is your impression of how the children and parents perceived and used the Apps?
    a. Based on your experience, what encouraged people to use the Apps regularly?
    b. Based on your experience, what discouraged people from using the Apps regularly?
    c. Have you observed differences between certain groups of patients? If yes, can you think of possible explanations?
4. What impact did the Apps have on how the children and their parents communicated with your team, e.g., were there changes in the way they reported their symptoms?
5. How frequently have you checked the answers of the patients?
    a. What impact did the Apps have on your own communication with the children and their parents?
    b. What impact did the Apps have on the communication within your team?
6. In your opinion, how do these eHealth solutions influence care? How else can these make a valuable contribution?

S5: Additional Information on Secondary Endpoints And ePRO-based Measures

Table S5: Overview of secondary endpoints and ePRO-based measures.

QUESTIONNAIRE	DIMENSIONS/SCALES/FACTORS	RATING SCALES OF ITEMS	SCORE COMPUTATION
**SSPedi/Mini-SSPedi**	15 cancer- and treatment-related symptoms:<br>Feeling disappointed or sad<br>Feeling scared or worried<br>Feeling cranky or angry<br>Problems with thinking or remembering things<br>Changes in how your body or face look<br>Feeling tired<br>Mouth sores<br>Headache<br>Hurt or pain (other than headache)<br>Tingly or numb hands or feet<br>Throwing up or feeling like you may throw up<br>Feeling more or less hungry than you usually do<br>Changes in taste<br>Constipation<br>Diarrhea	5-point Likert scales from 0 (*not at all bothered*) to 4 (*extremely bothered*)	Not applicable.
**PedsQL^{TM} 3.0 Cancer Module**	8 dimensions:<br>Pain and hurt<br>Nausea<br>Procedural anxiety<br>Treatment anxiety<br>Worry<br>Cognitive problems<br>Perceived physical appearance<br>Communication<br><br>Total score	5-point Likert scale from 0 (*never*) to 4 (*almost always*)	Items are recoded reversely, linearly transformed to a 0–100 scale, scores computed as mean values per dimension and a total score.
**EORTC PATSAT-C33**	Multi-item scales:<br>Technical Skills (Physicians)<br>Information Exchange (Physicians)<br>Affective Behavior (Physicians)<br>Information (HCPs)<br>Affective Behavior (HCPs)<br>Coordination<br>Interaction with HCPs<br><br>Single Items:<br>Family involvement<br>Access/parking<br>Access/way (ease of finding different departments)<br>Environment<br>Overall care	5-point Likert scale from 1 (*poor*) to 5 (*excellent*)	Multi-item scales are averaged and linearly transformed to a 0–100 range together with the single items.
**Impact on Family Scale**	4 factors:<br>Financial burden<br>Familial/social impact<br>Personal strain<br>Mastery<br><br>Total score	4-point Likert scale from 1 (*strongly agree*) to 4 (*strongly disagree*)	The items of the first three factors are recoded reversely and factor and total scores are computed by adding up the corresponding items' ratings.
**EuroQol EQ-5D-3L**	5 dimensions:<br>Mobility<br>Self-care<br>Usual activities<br>Pain/discomfort<br>Anxiety/Depression	Each dimension has three levels: 1 (*no problems*), 2 (*some problems*), and 3 (*extreme problems*). Visual Analogue Scale (VAS): rated between 0 (*worst imaginable health state*) to 100 (*best imaginable health state*)	5-digit-coding based on the ratings across the five dimensions.

HCPs = healthcare professionals, VAS = visual analog scale.

S6: Extended Results On Feasibility Of ePRO-Based Measures

Table S6.1: PedsQL^{TM} dimensional scores with significant results derived from the aligned rank transformation analysis.

DIMENSION	GROUP	SUBSAMPLES (*n*)	IMPUTED (*n*)	FACTOR TIME		FACTOR GROUP		INTERACTION TIMExGROUP	
				p	η_p^2	***p***	η_p^2	***p***	η_p^2
**Total Score**	Diagnosis	Leukemia = 6<br>Solid tumor = 18	5	**0.028**	**0.128 ****	0.242	0.062	0.291	0.055
	KPS	KPS ≥ 80 = 14<br>KPS < 80 = 9	5	**0.021**	**0.143 *****	0.823	0.002	0.357	0.050
**Nausea**	KPS	KPS ≥ 80 = 14<br>KPS < 80 = 9	5	**0.042**	**0.121 ****	**0.041**	**0.184 *****	0.175	0.075
**Treatment anxiety**	KPS	KPS ≥ 80 = 14<br>KPS < 80 = 9	5	0.189	0.072	**0.040**	**0.186 *****	0.072	0.104
**Procedural anxiety**	Diagnosis	Leukemia = 6<br>Solid tumor = 18	5	0.124	0.083	**0.040**	**0.178 *****	0.653	0.024
	Age	6–9 years = 9<br>10–13 years = 5<br>14–17 years = 10	5	0.067	0.107	**0.026**	**0.294 *****	0.440	0.086
	KPS	KPS ≥ 80 = 14<br>KPS < 80 = 9	5	**0.018**	**0.147 *****	0.130	0.106	0.100	0.094
**Worry**	Birth sex	Female = 16<br>Male = 8	5	**0.025**	**0.131 ****	0.774	0.004	0.738	0.019

KPS = Karnofsky Performance Status Scale, * indicates a small effect size η_p^2, ** indicates a medium effect size η_p^2e, *** indicates a large effect size η_p^2.

Table S6.2: PATSAT scales with significant results derived from the aligned rank transformation analysis.

SCALE	GROUP	SUBSAMPLES (*n*)	IMPUTED (*n*)	FACTOR TIME		FACTOR GROUP		INTERACTION TIMExGROUP	
				p	η_p^2	p	η_p^2	p	η_p^2
**Overall Care**	Birth sex	Female = 12<br>Male = 7	6	0.192	0.088	0.542	0.022	**0.044**	**0.145 *****
**Information Exchange (with physicians)**	Birth sex	Female = 12<br>Male = 7	6	**0.018**	**0.178 *****	0.481	0.030	0.575	0.038
	Age group	6–9 years = 7<br>10–13 years = 5<br>14–17 years = 7	6	**0.042**	**0.155 ****	0.964	0.005	0.613	0.086
	Diagnosis	Leukemia = 6<br>Solid tumor = 18	5	**0.039**	**0.150 *****	0.715	0.008	0.438	0.051
	KPS	KPS ≥ 80 = 14<br>KPS < 80 = 9	5	**0.012**	**0.190 *****	0.146	0.120	0.526	0.042
**Information Exchange (with nurses)**	Age group	6–9 years = 7<br>10–13 years = 5<br>14–17 years = 7	7	0.081	0.130	0.375	0.115	**0.024**	**0.253 *****
**Access/way**	Age group	6–9 years = 7<br>10–13 years = 5<br>14–17 years = 7	5	0.131	0.110	**0.046**	**0.141 *****	0.566	0.116

KPS = Karnofsky Performance Status Scale, * indicates a small effect size η_p^2, ** indicates a medium effect size η_p^2, *** indicates a large effect size η_p^2.

Table S6.3: IOFS factors with significant results derived from the aligned rank transformation analysis.

FACTOR	GROUP	SUBSAMPLES (*n*)	IMPUTED (n)	FACTOR TIME		FACTOR GROUP		INTERACTION TIMExGROUP	
				p	η_p^2	***p***	η_p^2	***p***	η_p^2
**Total score**	Age group	6–9 years = 7<br>10–13 years = 5<br>14–17 years = 7	5	0.217	0.088	0.974	0.003	**< 0.001**	**0.388 *****
**Financial burden**	Age group	6–9 years = 7<br>10–13 years = 5<br>14–17 years = 7	5	0.094	0.124	0.592	0.063	**0.039**	**0.233 *****
**Familial/Social impact**	Age group	6–9 years = 7<br>10–13 years = 5<br>14–17 years = 7	5	**0.029**	**0.170 *****	0.603	0.061	**0.022**	**0.256 *****
**Personal Strain**	Age group	6–9 years = 7<br>10–13 years = 5<br>14–17 years = 7	5	0.682	0.030	0.690	0.045	**0.019**	**0.262 *****

KPS = Karnofsky Performance Status Scale, * indicates a small effect size η_p^2, ** indicates a medium effect size η_p^2, *** indicates a large effect size η_p^2.

S7: Extended Results Of Focus Groups

Statements represent paraphrased and thematically coded excerpts from focus group transcripts.

S7.1: Focus Groups With Children And Parents

Coding System with Frequencies Of Linked Statements

CATEGORY	N	%
**1) Consequences of App Usage**	30	3.9
**1a) Benefits**	20	2.6
**1b) Consequences**	10	1.3
**2) Compliance**	18	2.3
**3) ePROs**	80	10.3
**4) Influences on Motivation**	45	5.8
**4a) User Experience**	27	3.7
**5) Communication**	20	2.6
**5a) Family Communication**	17	2.2
**5b) HCP-Patient communication**	57	7.3
**6) Recommendations**	100	12.8
**7) Multidisciplinary Approach**	5	0.6
**8) Expectations**	14	1.8
**9) Game Design**	81	10.4
**10) Impression**	18	8.9
**Positive**	105	2.3
**Negative**	63	13.5
**Neutral**	69	8.1
**TOTAL**	**779**	**100**

Paraphrased Statements

<u>Game Design</u>

It wasn't going anywhere. It was repetitive. There were no new levels. No need to be afraid of losing, nothing could happen to you, you couldn't even die. (Patient 1, FNBRNO)

<u>ePROs</u>

When I was hospitalized and had to process a lot, it was helpful for me and I used it often, because these questions fit at the time. It helped me process it all, both in the hospital and at home. (Patient 1, USAAR)

My child would rate his health better than it really was. He indicated that some symptoms weren't that bad, although they were perhaps worse than he would have admitted. (Parent 2, USAAR)

My child was answering optimistically even though he was having strong symptoms. Not at all matching my observations. I let him answer according to his perception. If in my opinion he was not doing well, I would report it as such separately. (Parent 3, MHH)

The App and the questions made me more sensitive and made me pay more attention to things that I wouldn't have otherwise thought of or that could easily be overlooked. (Parent 4, MHH)

It was quite good for my child to think about such things, to really listen to oneself and not always answer „everything is fine". (Parent 2, MHH)

The questions were unfitting at times, and my child couldn't really make sense out of it. Small children don't get bothered about such questions. (Parent 1, FNBRNO)

<u>Consequences of App Usage</u>

You need to find the time to concentrate and be truthful. If I received a notification while we are going through a difficult time, I used the opportunity to write things down. It helped, especially at the beginning of the treatment. (MHH, Parent 3)

<u>Benefits</u>

I liked to be sensitized to some issues and things that happen in everyday life that don't get forgotten because they got noted down. (Parent 2, MHH)

You're on a different level when you're playing and answering, and I think that's good that you don't have to really concentrate and think too profoundly when you are answering. The answers come naturally, from your interior. The answers come from within and not from thinking about what I wanted to say or how I wanted to express it to the doctor. That's gone. (Parent 3, MHH)

I always had the feeling that I was being listened to. I always had someone to talk to. We never felt like we were on our own. Maybe the App contributed to that feeling. (Parent 3, MHH)

The App gives children the opportunity to distract themselves and to deal with questions on how they feel without having the typical conversation with the doctor, to express themselves in another context. (Parent 3, MHH*)*

The App didn't have a significant effect on me. Just when my mood was off, it made me become aware of it, I would say. (Patient 1, MHH)

I would say that the effect is interdependent. That alone the fact that I'm asked those questions makes me become more sensitive about my child's wellbeing. The effect of the App is not uncomforting; it is minimal and rather positive. (Parent 4, MHH)

<u>Compliance</u>

I tried to use the App at least once weekly. However, when I noticed mood changes or deteriorations in my child I used it more frequently. (Parent 1, MHH)

I tried playing once a week, or during my medical appointment. (Patient 2, FNBRNO)

I used the App whenever I had some spare time on the bus, train or in the car. (Patient 3, USAAR)

With the App I could stay right there on the tablet and briefly answer questionnaires and then continue playing. (Patient 2, MHH)

My child used it mostly on the ward during waiting times when there was nothing to do. At home there was always something else to do. (Parent 2, USAAR)

It was a pity that the doctors didn't react to my reports. If they did, you would have all the more reason to use the App. (Patient 1, MHH)

I would have used the App more regularly if there had been consequences with the follow-up medical appointments. Otherwise, it was only for self-reflection, but the reach of the App is broader. (Parent 2, MHH)

I knew the MyPal team was expecting my involvement. They regularly checked-up on us and that also worked as a reminder that I had to use the App. I would have perhaps forgotten otherwise. (Parent 3, MHH)

It was more like a to-do I had on my list, until I got a call from a MyPal Team member because I had reported a symptom conspicuously often. That is when I realized there is a feedback loop. [...] I had understood the purpose of the App and this purpose became tangible when I got the call. (Parent 4, MHH)

<u>Influences on Motivation</u>

We were not just doing it for ourselves, but to benefit others through our participation in the study. (Parent 3, MHH)

<u>User Experience</u>

Something positive was that you could also use the App without the internet, and I could play in the car or while on the train. (Patient 3, USAAR)

I don't enjoy reading very much, so I liked the read-aloud function [of the questions]. (Patient 2, MHH)

<u>Communication</u>

I like that you can just write something down or express your feelings without having to talk directly to someone, it's kind of anonymous. (Patient 3, USAAR)

The App is a safe place, where you can tell the truth. In the App I can give free rein to my feelings. (Patient 3, USAAR)

It was better for my child to share things through the App. For me, it was the same. I would share through the App things I wouldn't share face to face. When something was worrying me at a specific moment, I got it out of my chest and moved on. (Parent 3, FNBRNO)

Unlike my child, an introverted child would feel more comfortable sharing things through the game. (Parent 1, FNBRNO)

As a patient, the App is an easy way to talk about your problems, for me easier than doing it face-to-face. (Patient 2, MHH)

Family Communication

I couldn't detect much of a difference with the App. We often talk about the state of health anyway, I couldn't say that the App has had an impact there. (Parent 1, USAAR)

Our communication does not depend on the App. We communicate about the fears and griefs we are currently going through. However, I reported stronger feelings like aggression freely in the App. Privately we talked differently. (Parent 1, MHH)

I think it's better this way than speaking to my mum about it for 2 hours. (Patient 3, MHH)

My child would ask me sometimes how to proceed, if there were any doubts on how to answer. (Parent 2, USAAR)

Sometimes I would sit down with my mum, and we did it together, we filled it out, because I knew more than she did, I knew best, what was hurting me. (Patient 2, FNBRNO)

As a consequence of the diagnosis, we now talk differently about health, with or without an App. I can´t be certain that this difference was only due to the App. (Parent 4, MHH)

HCP-Patient Communication

The doctors did ask me if I had used the App, but my reports were not discussed. (Patient 4, MHH)

The lack of reactions from HCPs was disappointing. (Patient 1, MHH)

It was a pity that the doctors didn't react to my reports. If they did, you would have all the more reason to use the App. (Patient 1, MHH)

The fact that you need to attend the hospital frequently is enough, you don't need to share things via App. It is all covered by the personal visits. (Patient 2, FNBRNO)

I would have answered if someone had asked. But it didn't necessarily have to be that way. (Patient 3, USAAR)

In the doctor's consultation, the questions they make are in part redundant to the questions you get in the App. But it makes sense to talk about them again in person during appointments. (Patient 4, MHH)

Impression

So, the concept and the idea were great, it's just the way it was carried out. (Parent 1, FNBRNO)

For MyPal to work, you definitely have to try to get the whole clinical staff on board. A real exchange will only be possible if everyone is involved. (Parent 2, MHH)

Recommendations

It would be great if the information that we entered into the App would also flow into other specialty departments and not just oncology. Maybe markers could be added to help identify when to start certain therapies or when to refer to another specialty. (Parent 4, MHH)

It would be better to involve the entire staff of the clinic, the doctors and the psychosocial service. If this is not possible, there should be a MyPal team on site that is informed about everything, like in this case. (Parent 3, MHH)

It would be cool if you could make something with the artifacts you collect, like building a house. (Patient 1, FNBRNO)

By sharing the game with other children who have the illness, it builds a sort of community. (Parent 3, MHH)

A function to enable communication with other parents and patients would be helpful. We are all going through the same and you should be able to talk to others who are in a similar situation. (Parent 1, MHH)

S7.2: Focus Groups With Project-Internal HCPs

Coding System With Frequencies Of Linked Statements

CATEGORY	N	%
**1) Consequences of App Usage**	13	3.0
**1a) Benefits**	17	3.9
**2) Compliance**	33	7.6
**3) ePROs**	14	3.2
**4) Influences on Motivation**	64	14.8
**4a) User Experience**	20	4.6
**5) Communication**	4	0.9
**5a) HCP-Patient Communication**	37	8.6
**6) Recommendations**	23	5.3
**7) Multidisciplinary Approach**	0	0
**8) Expectations**	19	4.4
**9) Game Design**	15	3.5
**10) Impression**	20	4.6
**10a) Impression of eHealth Solutions**	16	3.7
**Positive**	55	12.7
**Negative**	61	14.1
**Neutral**	21	4.9
**TOTAL**	**432**	**100**

Paraphrased Statements

Game Design

They enjoy games that are competitive. They didn't find the purpose of this game; it was not catchy enough. (Physician, USAAR)

Expectations

It would be interesting to see if the children would use and benefit from ePROs. Would it help them deal with the disease? What are the benefits for the HCPs? Would the parents be supportive? (Physician, USAAR)

I had questions rather than expectations: how will it work? What are patients going to report to us? How are families going to deal with this? Is there skepticism? Can we establish this here in the clinic? Will it be accepted? (Physician, MHH)

ePROs

We had some patients that told us they didn't use the App because it reminded them of their disease. They didn't want to have anything to do with the hospital as long as they were at home. (Physician, MHH)

Consequences of App Usage

It didn't have an impact on the communication of the team; it was just an addition to the things we do directly. (Physician, MHH)

Benefits

Teenagers would write down things that they might not have brought up in front of their doctor. It offered them an easier path to let things out. [...] They use it to avoid interactions, uncomfortable or intimate situations, especially if they are prone to shyness or anxiety. (Psychologist, MHH)

Being in follow-up care gives you more certainty. You have less acute problems, the burden drops, you have more freedom. And in the background you have this possibility to make reports and communicate, to stay in contact. (Psychologist, MHH)

When I reported something that a patient had notified through the App, the outpatient clinic was actually very grateful for it. All my reports were well received. [....] I remember a patient, a teenager, who reported something he didn't want to report face-to-face. He thanked me afterwards. The feedback that I got was always positive. (Nurse, MHH)

If I am already being treated acutely and I am there every other day, I don't need to over report my status. (Psychologist, MHH)

From the patient's point of view, it is different because you're involved in your diagnostic and treatment process, you have a completely different ability to act, your agency is completely different. It is a communication channel. It empowers. (Psychologist, MHH)

<u>Influences on Motivation</u>

Using the diary function was motivating. They used it either to share something with us or to write something from the depths of their souls. (Psychologist, MHH)

The return on investment is rather low. They are already overloaded, then they are supposed to do a lot and then they do not get much in return. It becomes a cost-benefit issue. Should I keep investing energy? It became a minor priority. (Psychologist, MHH)

App usage depended on many factors, e.g., we reminded them regularly, we showed presence to let them know that the App has a purpose. That if you make a report, it's going to get somewhere, to someone, to the HCPs. (Psychologist, MHH)

I could have reminded the patients to use the App, but I think it would bias the results. They should use it on a voluntary basis, the motivation should be different. (Physician, USAAR)

At some moments, when we need most of the information from them, they are often so tired that they don't feel like playing the game. (Physician, FNBRNO)

We had families who didn't agree to participate in the study, not because they didn't like the idea, but for other reasons: the resources were not available, or they were overwhelmed. There is too much strain and uncertainty shortly after the diagnosis. (Psychologist, MHH)

Boys tend to play and gamble more. And girls, especially teenagers, are more prone to share emotion and social information. Communicating or being socially compliant because they are participating in a study. In that respect there could be a gender effect (Psychologist, MHH

I noted differences between patients in follow-up vs acute care. Follow-up patients tended to be more diligent. Perhaps they were doing a lot better or had more free time. However, most of them were girls. Is it an effect of health-status or of gender? (Physician, MHH)

When you have something new, it is still inefficient, it comes with a risk, so people tend to use the tools they already are familiar with, because they are more reliable to use. (Psychologist, MHH)

They knew that the MyPal team would read the patient notifications and would report if something was off. They knew that really life-threatening things wouldn't be shared through the App. (Physician, MHH)

Because of the pandemic, there were staff shortages. For example, the nurses were worried that with MyPal they would be assuming an extra responsibility that would cost them time. So, they didn't want to get involved. At first the nurses were in favor, then it became a time management issue. They are working overtime and have to cover the shifts of sick colleagues. The project was already running, and they thought, if I already missed the start, why should I begin now? (Nurse, MHH)

<u>Communication</u>

When the communication within all team members and with the patients and their parents is already working optimally, then such Apps become secondary. (Physician, USAAR)

HCP-Family Communication

Normally the patients have to report via phone call. In particular cases, if the patient reported something through the App some of the staff at the clinic would tell them to report things by phone, not through the App. In our case, it was counterproductive. (Physician, MHH)

The App gave us material for conversation. We could ask about their symptoms and their context. It allowed us to develop better communication with the patients and parents. They knew we were reading their notifications; we were keeping up. (Physician, MHH)

We had something tangible. We would visit them with our tablet, we could see their reports, show them their progress. We had a basis for communication. It made things easier, a conversational anchor. (Psychologist, MHH)

The communication between HCP remained the same. When the communication within all team members and with the patients and their parents is already working optimally, then such Apps become secondary. If the communication is not working optimally, such Apps could be helpful for children and their parents. I think this also has an influence on the motivation to use it. If the clinic offers good communication, then this is less important. (Physician, USAAR)

Impression of eHealth Solutions

It makes sense if it is supplementary and not substitutive. If it's complementary, it has its place and its context. (Psychologist, MHH)

eHealth is making an impact, as a fact. In politics, they talk about money to fund Apps, as they are perhaps more profitable. It is also a big research topic. The question is how to assure their quality. The market is large. (Psychologist, MHH)

It should become a tool for certain situations. We can't get around eHealth, we live in a digital world. But the quality with which it has to be done is yet to be defined. (Physician, MHH)

I am not sure that healthcare can really be improved with eHealth solutions. Parents and children will continue to make phone calls, regardless of such Apps. (Physician, USAAR)

It definitely has an impact, but in which direction? One would desire that the impact is favorable. But we have seen that if I communicate and nothing comes back, it can have the opposite effects. It depends on the conditions. (Psychologist, MHH)

Why should I install this App on my private mobile? (keep working and private life apart from each other. (Nurse, MHH).

Recommendations

Sensors, pedometers, pulsometers, and such could give us information that you don't usually get from the patient directly [through self-reports]. These could motivate the HCP team to act. (Psychologist, MHH)

Could you get information through other methods where the patient does not have to actively report something: (e.g., pain = heart rate increases)? The patient plays and we get information indirectly. E.g., counting steps to determine physical activity. (Physician, USAAR)

For research purposes it is important to create prediction models out of direct data, but also data out of social networks, step counts, pulse, wearables, is needed to make big data prediction models. E.g., If you could forecast pain, you could act up front (Psychologist, MHH)

S7.3: Focus Groups With Project-External HCPs

Coding System With Frequencies Of Linked Statements

CATEGORY	N	%
**1) Consequences of App Usage**	9	3.7
**1a) Benefits**	1	0.4
**2) Compliance**	10	4.1

**3) ePROs**	4	1.7
**4) Influences on Motivation**	26	10.7
**4a) User Experience**	19	7.8
**5) Communication**	10	4.1
**5a) HCP-Patient Communication**	15	6.2
**6) Recommendations**	27	11.1
**7) Multidisciplinary Approach**	3	1.2
**8) Expectations**	9	3.7
**9) Game Design**	15	6.2
**10) Impression**	15	6.2
**10a) Impression of eHealth Solutions**	11	4.5
**Positive**	27	11.1
**Negative**	26	10.7
**Neutral**	16	10.7
**TOTAL**	**243**	**100**

Statements

Game Design

The market these days is full of good video games and as such it's tricky. (Physician, FNBRNO)

Expectations

That it would be something where they don't mindlessly consume a game or video but do something meaningful with it. Children who don't know what to do with it probably wouldn't have used it anyway. So, we wouldn't have driven anyone into media consumption with it. (Psychologist, MHH)

Consequences of App Usage

There was also a phase when I had a few patients for whom this was helpful because they sometimes entered something that I was looking after, especially in the outpatient clinic. [...] Otherwise it hasn't changed anything for my psychological work area, our social workers haven't actually used this App, so it doesn't seem to have any significance for their work either. And the nursery teacher didn't use it either. (Psychologist, MHH)

One family once said that there were some symptoms, but they couldn't remember exactly. Then they took out the App and looked up for themselves when it actually happened. They were then able to sort it out a bit for themselves. (Physician, MHH)

No, it didn't have any impact on daily practice. (Physician, FNBRNO)

Compliance

I've often had patients ask me specifically about a symptom and say: 'I've entered that too.' Then I looked it up. Otherwise, to be honest, the inhibition threshold was quite high, I simply didn't do that in everyday life, especially not in this routine of seeing acute outpatient children. (Psychologist, MHH)

There was no data to check, as the kids didn't play it. Therefore, I must admit I wasn't that active. (Physician, FNBRNO)

It would probably have been less time-consuming if you had been in there more often. Because then you would have had more automatisms. It was time-consuming that way, if you're not in for a while you have to think about how to get there, what my password was, what my username was again. That was time-consuming. Yes. (Physician, MHH)

Influences on Motivation

I imagine that the better the patients feel and the more active they can be, the fewer people use the App because they are sitting or lying around less and the more time patients spend in bed or resting, the more time they spend using their tablets and mobile phones. (Physician, MHH)

Some children actually use tablets and mobile phones a lot in hospital and parents are happy that they are no longer so present at home. Maybe that's also because, nicely, other activities are available again and the mobile phone is no longer so attractive and no longer so present for some children. (Psychologist, MHH)

I have the feeling that parents who were particularly interested in keeping a close eye on their children and also keeping a close eye on what is happening during the time they are at home so that they can make the best possible use of it for the therapy, so that their children can also be looked after in the best possible way. That was an incentive for them to simply write down what they might otherwise have brought with them on a piece of paper. (Psychologist, MHH)

The older the users were, the more I had the feeling that it was less about playing and more about sharing information. (Physician, MHH)

User Experience

That I could look very quickly if something was conspicuous in the psychological area, are there any physical symptoms which occur together? [...] I actually found that a very practical update. (Psychologist, MHH)

Communication

I believe that exactly this kind of children, who are hooked on the Apps, they grow up on them, and it can be a more pleasant way for them to share their feelings. (Physician, FNBRNO)

HCP-Family Communication

There is added value if information is accessible in an easy way. I think I would actively ask certain questions in certain directions much more often if the patient directed me in this direction by describing their symptoms. Reports through the App could be used to guide the patient a little more towards certain issues that are perhaps being overlooked. (Physician, MHH)

If I meet them once and they tell me they're fine and then I meet them again in a few weeks and they're fine and that means everything is always okay. But if I saw an entry in the App that things were different for a while, then I could talk to them again and remind them: 'Look, you entered something there, what was going on?'. Then we would be even more likely to get in touch again about an unpleasant topic. (Psychologist, MHH)

An interaction is something else when I can personally correct something, meaning that something develops, or when I can personally ask again how things were meant. When you answer, you have to decide, for example on a categorization or something. And it only becomes clear in the conversation what is really meant by it. That is something that perhaps cannot be grasped in this way [i.e., the App]. (Psychologist, MHH)

Impression of eHealth Solutions

I think that nowadays, digitalization makes sense and we're heading there in all areas of activity. I personally see a big boom of these things in medicine in general. (Physician, MHH)

I see that e-medicine and e-counselling are really in nowadays, which I think is very useful to reduce the burden of emergency offices. You can discuss some things online instead of coming to the hospital in person with less severe issues. All in all, yes, it's a way, in my opinion. (Physician, FNBRNO)

In general, I do believe that many of the e-approaches improve medical care. In terms of content, but also in terms of organization. There are certainly Apps that remind you of appointments, for example, or that allow you to simply scan and send findings and know where to send them. (Physician, MHH)

It must be complementary, not substitutive. I couldn't imagine it any other way. Because with how many families do we need an interpreter to communicate with our hands and feet, they wouldn't be able to use it at all. They would no longer have access. (Psychologist, MHH)

You have to go through a lot of places again to really be able to use it and we would have to function much more digitally overall to really be able to use these e-health applications such that every patient is able to install an App on their mobile phone. I think that's a really big issue. Even if we could, the MHH would first have to say that it really is a programme that is allowed to run. So, there are also the administrative and data protection hurdles. They're insanely high. (Physician, MHH)

Both sides must have good access to it. I believe that the problematic side is less the patient side than the healthcare provider side. (Physician, MHH)

As with some other things, it would have to become established. And that means it has to be uncomplicated. As uncomplicated as possible. (Psychologist, MHH)

The flow of information in such large teams is quite difficult...well, I also experience this with other things, and it has to be repeated regularly, if it is not part of everyday life for the respective professional groups, it is not automatically taken into account during induction. (Psychologist, MHH)

Recommendations

Or these types of games where you build your own town every day. You just have to visit the App every day, because you collect money from the houses you've built. If you don't collect it, you lose it. So, something of this kind, which makes you open the App at least once a day, even if it was for 5 minutes. (Physician, FNBRNO)

Tamagotchi seems like a great idea also because it can become sort of a buddy to you for the treatment. The questions on a health state, which, at the current App, were emerging anonymously from somewhere deep down, would be asked by your Tamagotchi. And you get a digital during the treatment. All we want from the App could be delivered by this virtual avatar, which you can also create, customize, etc. (Physician, FNBRNO)
